# Investigating seasonal air quality variations consequent to the urban vegetation in the metropolis of Faisalabad, Pakistan

**DOI:** 10.1038/s41598-023-47512-y

**Published:** 2024-01-03

**Authors:** Muhammad Azeem Sabir, Muhammad Farrakh Nawaz, Tanveer Hussain Khan, Usman Zulfiqar, Fasih Ullah Haider, Abdul Rehman, Irfan Ahmad, Fahad Rasheed, Sadaf Gul, Safdar Hussain, Rashid Iqbal, Talha Chaudhary, Abd El-Zaher M. A. Mustafa, Mohamed S. Elshikh

**Affiliations:** 1 Institute of Forest Sciences, The Islamia University of Bahawalpur, Bahawalpur, 63100 Pakistan; 2 Institute of Environmental Studies, University of Karachi, Karachi, Pakistan; 3 Department of Agronomy, Faculty of Agriculture and Environment, The Islamia University of Bahawalpur, Bahawalpur, 63100 Pakistan; 4 Key Laboratory of Vegetation Restoration and Management of Degraded Ecosystems, South China Botanical Garden, Chinese Academy of Sciences, Guangzhou, 510650 China; 5 University of Chinese Academy of Sciences, Beijing, 100039 China; 6 Department of Forestry & Range Management, University of Agriculture Faisalabad, Faisalabad, Pakistan; 7 Department of Botany, University of Karachi, Karachi, Pakistan; 8 Department of Forestry and Range Management, Kohsar University Murree, Murree, Pakistan; 9 Faculty of Agricultural and Environmental Sciences, Hungarian University of Agriculture and Life Sciences, Godollo, 2100 Hungary; 10 Department of Botany and Microbiology, College of Science, King Saud University, Riyadh, 11451 Saudi Arabia

**Keywords:** Environmental sciences, Biodiversity, Community ecology, Conservation biology, Ecophysiology

## Abstract

Urban atmospheric pollution is global problem and and have become increasingly critical in big cities around the world. Issue of toxic emissions has gained significant attention in the scientific community as the release of pollutants into the atmosphere rising continuously. Although, the Pakistani government has started the Pakistan Clean Air Program to control ambient air quality however, the desired air quality levels are yet to be reached. Since the process of mapping the dispersion of atmospheric pollutants in urban areas is intricate due to its dependence on multiple factors, such as urban vegetation and weather conditions. Therefore, present research focuses on two essential items: (1) the relationship between urban vegetation and atmospheric variables (temperature, relative humidity (RH), sound intensity (SI), CO, CO_2_, and particulate matter (PM_0.5_, PM_1.0_, and PM_2.5_) and (2) the effect of seasonal change on concentration and magnitude of atmospheric variables. A geographic Information System (GIS) was utilized to map urban atmospheric variables dispersion in the residential areas of Faisalabad, Pakistan. Pearson correlation and principal component analyses were performed to establish the relationship between urban atmospheric pollutants, urban vegetation, and seasonal variation. The results showed a positive correlation between urban vegetation, metrological factors, and most of the atmospheric pollutants. Furthermore, PM concentration showed a significant correlation with temperature and urban vegetation cover. GIS distribution maps for PM_0.5_, PM_1.0_, PM_2.5,_ and CO_2_ pollutants showed the highest concentration of pollutants in poorly to the moderated vegetated areas. Therefore, it can be concluded that urban vegetation requires a rigorous design, planning, and cost–benefit analysis to maximize its positive environmental effects.

## Introduction

Negative health impacts of air pollution are gaining significant attention as they are becoming more prevalent, as major urban cities are experiencing more frequent instances of elevated pollution^1,2^ due to increase in atmospheric emissions^3,4^. It is projected that by 2050, 66% of the global population will reside in urban areas^5^. In 2015, air pollution was responsible for 8 million deaths worldwide^6^. Therefore, air pollution has become a critical environmental concern in the present century, primarily driven by industrialization, urbanization, and the growth of road traffic. This issue is particularly prominent in developing nations like Pakistan^7,8^.

The atmosphere, which is combination of various gasses, plays a vital role in sustaining human life^9^. Among those gasses, Carbon dioxide (CO_2_) is commonly referred as a pollutant gas and constitutes approximately 0.03% of the air. The combustion of fossil fuels in modern times releases CO_2_ into the atmosphere^10^. This over production of CO_2_ is creating a layer around the Earth, which is causing the greenhouse effect and global warming. The repercussions of air pollution and consequently climate change can lead to decreased crop production and eventually food scarcity^11^. Annually, During the period of human evolution, the ambient atmospheric CO_2_ levels remained relatively stable, below 300 parts per million (ppm)^13^. However, currently, about 30 billion tons of CO_2_ is emitted into the Earth's atmosphere annually^12^. The United States Occupational Safety and Health Administration (OSHA) has established a permissible exposure limit (PEL) for CO_2_, at 5,000 parts per million (ppm) (or 0.5%) over an 8-h period^14^. Studies on the health effects of air pollutants have associated CO_2_ with symptoms like respiratory illnesses, including sneezing, rales, wheezing, rhinitis, and asthma^15,16^. Additionally, other symptoms such as coughing, headaches, and irritation of mucous membranes have also been identified^16^. Difficulties in concentration have been linked to CO_2_ levels exceeding 1000 ppm. Gaihre et al.^17^ discovered that CO_2_ levels above 1000 ppm are associated with decreased school attendance, and teachers have been reported with neuro-physiological symptoms like headaches and fatigue^18^.

The presence of urban vegetation can help mitigate the heat island effect and enhance the air quality in urban areas^19,20^. Incorporating trees within green infrastructure has proven effective in reducing particulate matter (PM) levels in urban environments^21,22^. Factors such as tree species, canopy size, structure, leaf area density, and the positioning of trees around buildings can influence the amount of particulate matter present^23^. Recent studies have extensively explored the deposition velocity and particulate capture efficiency of various tree species through wind tunnel experiments and field studies^24,25^. Moreover, research has also been conducted to investigate the impact of green roofs on PM deposition^26^.

The types of air pollution can vary from one location to another, influenced by factors such as human activities, topography, and other elements^27^. Meteorological conditions have a significant impact on air pollution levels and the direction of air movement^28^. Other meteorological factors also play a crucial role in the dispersion of air pollution, with their influence varying across seasons, as well as day and night periods^29^. Variables related to weather forecasting, such as rainfall, relative humidity, wind direction, wind speed, and temperature, affect the levels of PM_2.5_ and PM_10_, which are types of particulate matter^30^. Several studies have reported that the concentration of air pollutants varies based on meteorological factors^27^, the pollution sources, and local topography^31^. In typical urban environments, the population is exposed to approximately 200 different air pollutants or forms of air pollutants^32,33^. Among these pollutants, PM holds significant importance due to its high measured levels^34–36^. Concentrations of PM_10_, which refers to particulate matter with a diameter less than 10 μm, in areas such as Athens and Greece, have shown significant correlations with other pollutants and meteorological parameters like nitrous oxides (NOx), carbon monoxide (CO), and solar radiation^37^. Negative correlations have been observed between PM_10_ concentrations and secondary pollutant ozone (O_3_), wind speed, and precipitation^38^. PM_10_ has also demonstrated an inverse relationship with temperature and relative humidity (RH)^39^.

Particulate matter (PM) present in the atmosphere has significant implications for visibility, the formation of acid rain, climate change, and human health^40^. PM, with its varied sizes, poses a threat to all forms of life and serves as a crucial indicator of air pollution^41^. Since the era of industrialization, particular attention has been given to particles smaller than 2.5 μm (PM_2.5_) due to their detrimental effects on human health, as they can easily penetrate the lungs^42,43^. The majority of urban cities worldwide have PM_2.5_ levels exceeding the air quality standards set by the World Health Organization (WHO) and their respective countries^43^. Numerous reports have confirmed the negative impact of PM_10_ on health, linking airborne PM to premature mortality and various other health risks in cities across the globe^44,45^. On the other hand, a recent epidemiological study focusing on ultrafine particle mass discovered significant associations with premature mortality^46^. Despite years of progress, PM concentrations in many urban areas in the United States still surpass health-based standards, leading to an increase in non-accidental mortality^47,48^.

Above all, the adverse effects of airborne particulate matter (PM) on human health and the global climate have garnered significant public attention^49,50^. It is crucial to have a comprehensive understanding of the major sources of PM_2.5_ and their respective contributions in order to design effective strategies for reducing PM_2.5_ levels^51^. Several observational studies have investigated the correlations between airborne PM concentrations and meteorological parameters, although limited to small regional areas or specific pollutant species (such as PM particles, ozone, COx, SOx, and NOx) and specific meteorological conditions^52–54^. Lee and Hieu^55^ conducted an analysis of episodes with high PM_10_ concentrations in Korea, focusing on their association with meteorological conditions.

Maintaining good air quality is crucial for both human health and the environment, yet monitoring sources of contamination can often be challenging^35^. Geographic Information System (GIS) technology offers a valuable tool for managing statistical and spatial data, allowing us to understand the relationship between air quality and its impact on human and environmental well-being^56^. By utilizing GIS, it becomes possible to monitor pollutant emissions and track the effects of harmful airborne pollutants such as smog and dust on plant and human life, as regulated by the Environmental Protection Agency (EPA)^57^. Conservationists can leverage GIS to ensure that no further pollution occurs by monitoring these relationships and identifying the sources of pollutants^58^. GIS technology possesses the advantage of analyzing spatial data effectively and handling large spatial databases, which is particularly valuable for air pollution studies where significant amounts of data are involved, including air pollutants, wind direction, wind speed, traffic flow, solar radiation, and air temperature^59^.

Although much research has been done to evaluate the potential of urban vegetation and seasonal change on the air quality in the developed countries’ residential areas but the knowledge about the effect of urban vegetation and seasonal change on the air quality in residential areas of developing countries like Pakistan and its industrial city Faisalabad needs to be more extensive. Therefore, the primary objective of this study was to investigate the relationships between the concentration of atmospheric pollutants PM_0.5_, PM_1.0_, and PM_2.5_, sound intensity, CO, and CO_2_ pollutants with urban vegetation and metrological parameter. Moreover, GIS techniques have been employed to map the spatial distribution and dispersion of atmospheric pollutants in the Faisalabad area. These pollutant maps are valuable for establishing the appropriate placement of air pollution measurement stations, ensuring accurate monitoring and assessment of air quality.

## Materials and methods

### Study area

**Figure 1:**
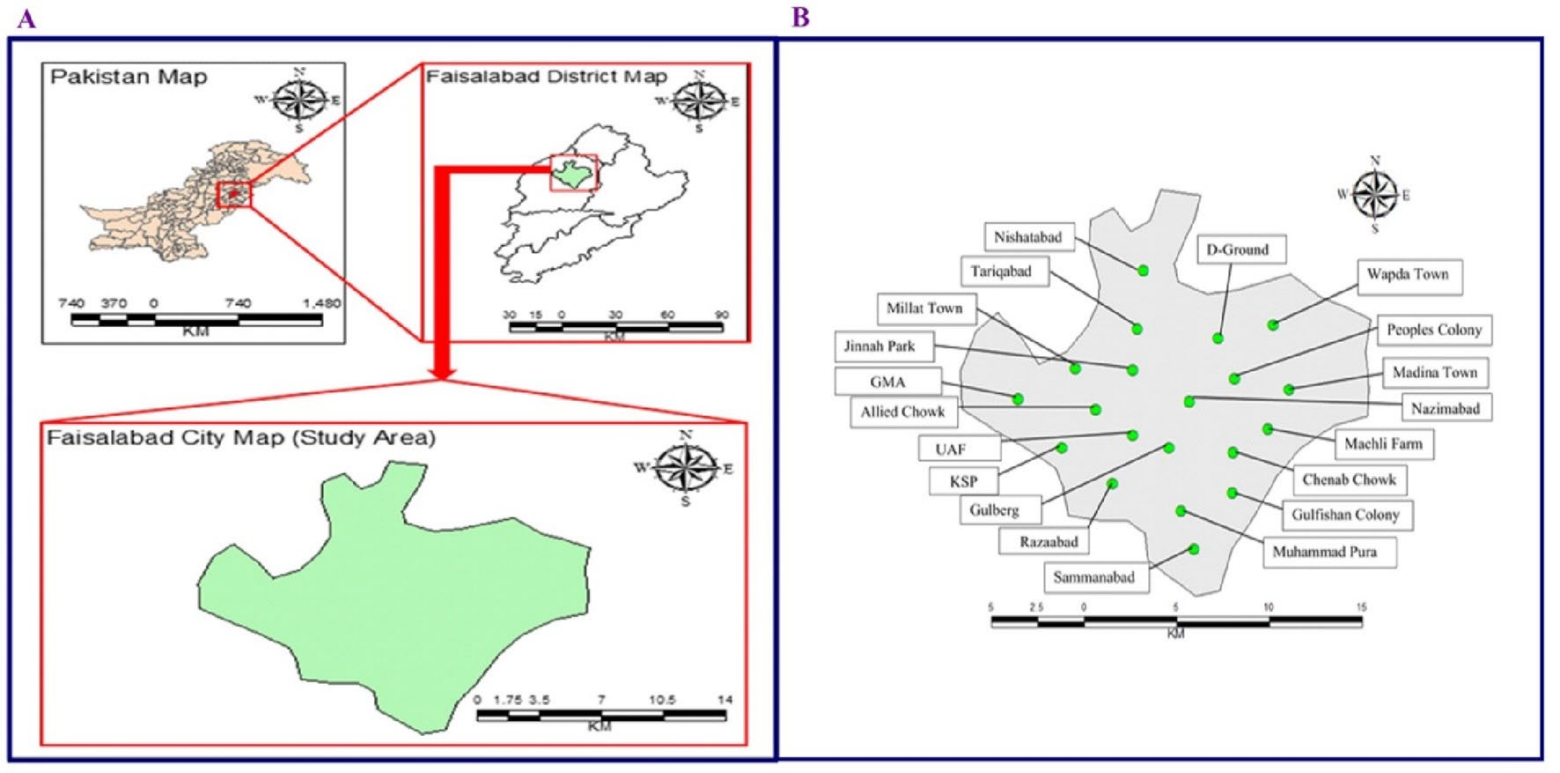
(**A**) Map of Pakistan and Faisalabad city and (**B**) Faisalabad city map with their twenty working sites.

The study focused on urban areas of the Faisalabad province of Punjab, Pakistan. The city is located in the flat plains of northeast Punjab, Pakistan (31°24/N, 73°04/E; Fig. 1A). Owing to the vast textile industry related to weaving, dyeing, printing, and finishing cloth Faisalabad city is rapidly becoming populous and resultantly facing acute problem of atmospheric pollution^60^. The overall climate dominates the subtropical climate with hot and humid summers and cool and dry winters. During the sampling period, average day/night RH was 33.1/75.1% and temperatures was 38.28 ± 4 °C and 22.82 ± 3.6 °C, respectively.

### Ground-base/field data measurements

Concentration of atmospheric pollutants were collected at 20 locations within the city during summer and winter seasons, respectively (Fig. 1B) using portable air quality monitor (Series 500, Aeroqual, Auckland, New Zealand). The sound intensity was measured using Sound Level Meter (ACO 6230, Qte Technologies, Vietnam). A portable weather station (RK900-01, RIKA, China) was used to measured temperature and relative humidity. Particulate matter concentration was measured in ug/m^3^, CO and CO_2_ concentration was measured in ppm, temperature (C), relative humidity in percentage (%), and sound intensity was measured in dB. Tree density and diversity was measured manually by counting the number of trees.

### Statistical analysis

Pearson correlation analysis was performed to describe the pattern of association between urban trees and atmospheric pollutant concentration parameters. Principal component analysis (PCA) based on the correlation matrix. All tests were performed using R Studio software. The maps were developed using ArcMap 10.3 and by using this software we have made the maps.

### Plant guidelines

All the experiments were done in compliance with relevant institutional, national, and international guidelines and legislations.

## Results

### Seasonal changes of particulate matter in an urban area

**Figure 2:**
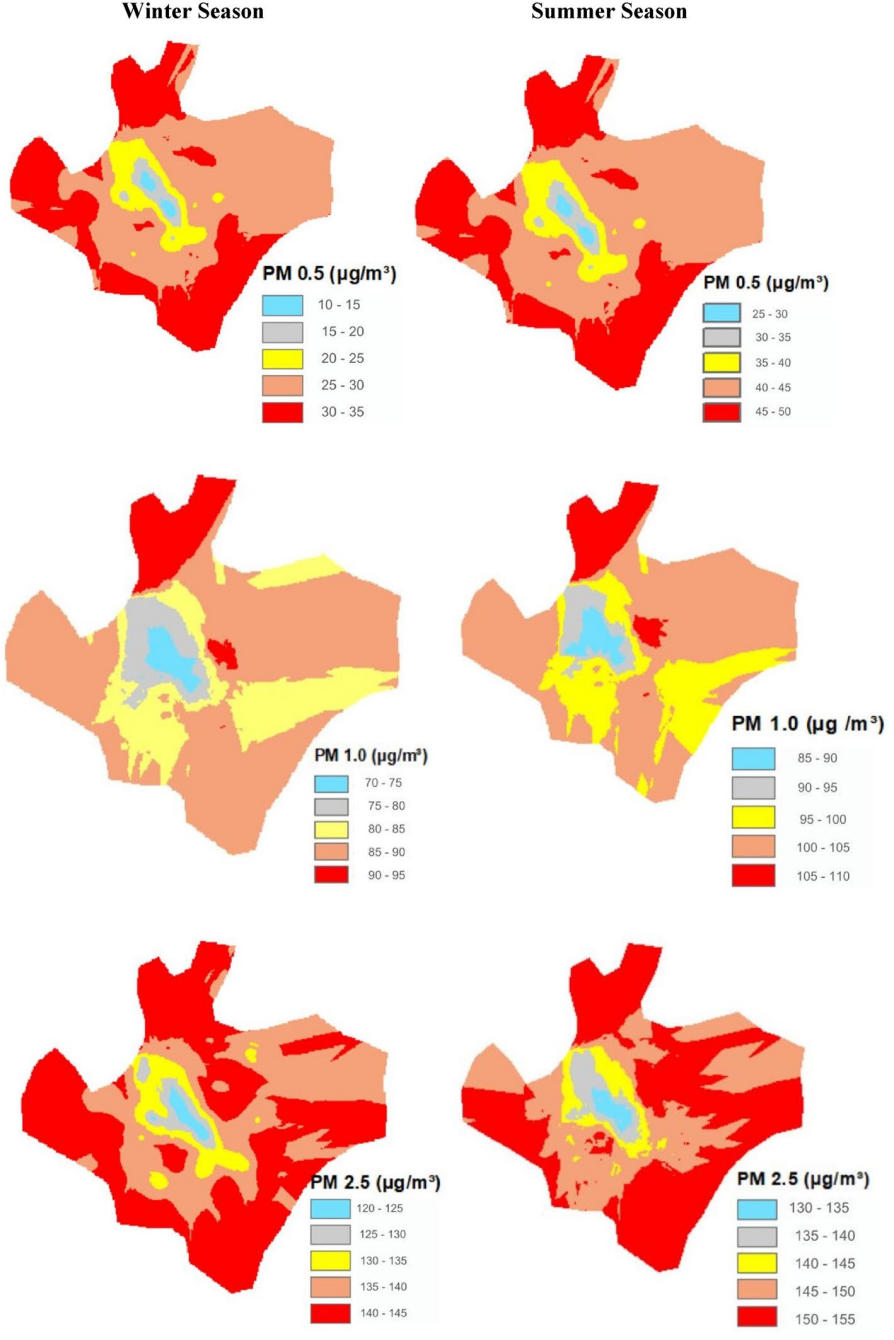
Effect of urban vegetation on particulate matter in Faisalabad city throughout winter and summer season.

The PM_0.5_ concentration was recorded in different seasons and found to vary from summer to winter season (Fig. 2). In the urban areas, the highest concentration of PM_0.5_ was found during the summer season. The humidity in the summer is quite low due to which the particulate matter freely moves in the atmosphere and dry surfaces. Also, vehicular combustion leaves the particle easily transported due to the current of air. During the summer season, concentration of PM_0.5_ ranged from 25 to 50 µg m^−3^ particularly towards the periphery of the study area. The range of PM _0.5_ declined in the winter season and varied between 10 and 35 µg m^−3^. Similarly, the concentration of PM_1.0_ µg m^−3^ varied between 85 to 110 µg m^−3^ during the summer and between 70 to 95 µg m^−3^ during the winter season. The highest concentration of PM_2.5_ was recorded in the summer season which was between 130 and 150 µg m^−3^. During winter season concentration of PM_2.5_ ranged from 120 to 145 µg m^−3^. The concentrations of particulate matter varied in both the size from fine to coarse and also declined from summer season to winter seasons for three types of PM_0.5_, PM_1.0_, and PM_2.5_.

### Analysis of CO_2_ changes to vegetation cover in the city

**Figure 3:**
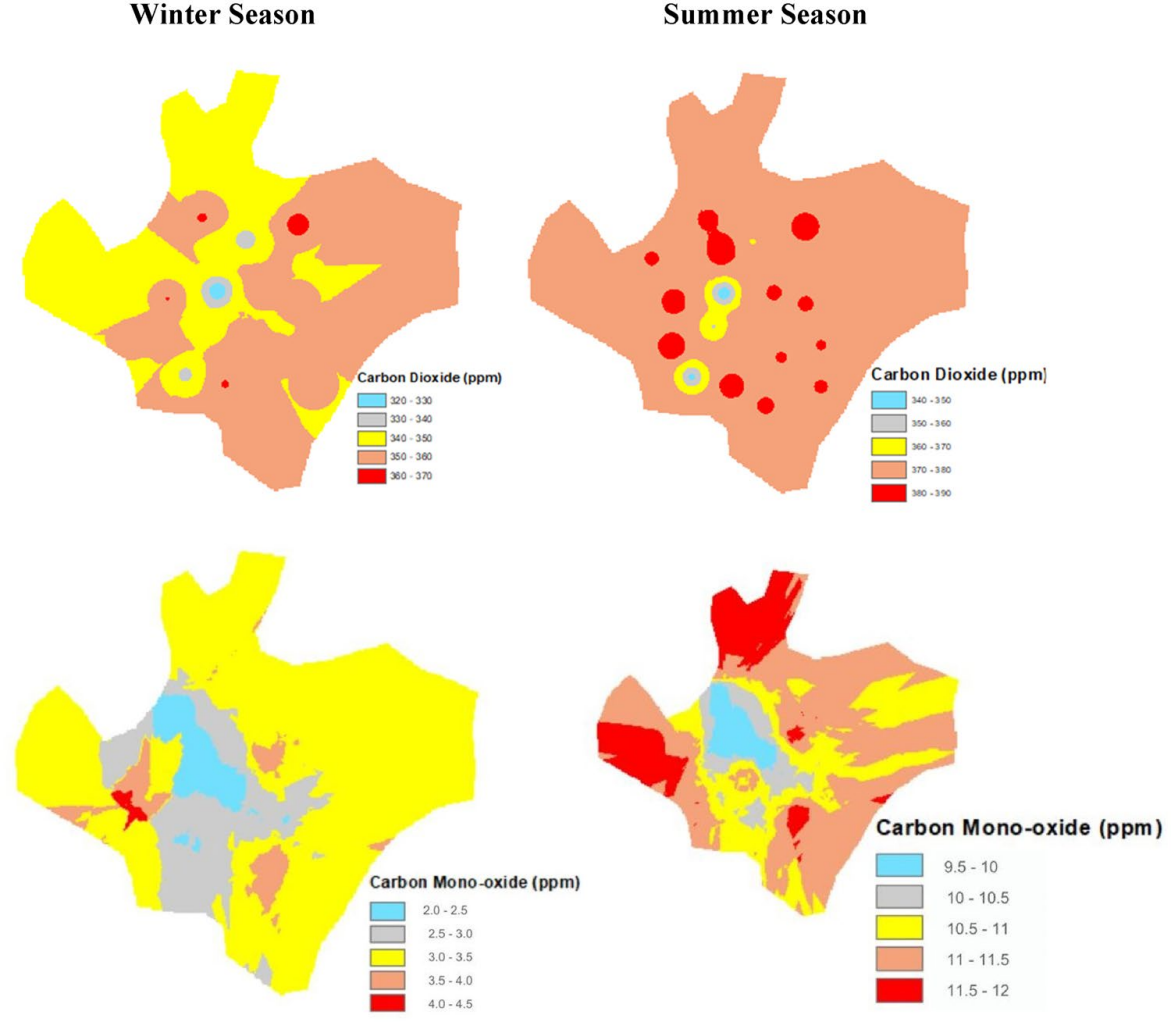
Effect of urban vegetation on carbon dioxide and carbon monoxide concentration in Faisalabad city throughout winter and summer season.

The CO_2_ and CO concentrations were recorded in the summer and winter seasons all over Faisalabad city. The highest CO_2_ concentration was found at 390 ppm and a lower was evidenced of 340 ppm. In the summer season, the CO concentration of 9.5 ppm to 12 ppm in the city was lower than the CO_2_ concentration. In the winter season, the concentration of 2.0 ppm to 4.5 ppm. The detail is given in Fig. 3.

### Temperature mitigation of green space, and humidity in the urban seasons of Faisalabad

**Figure 4:**
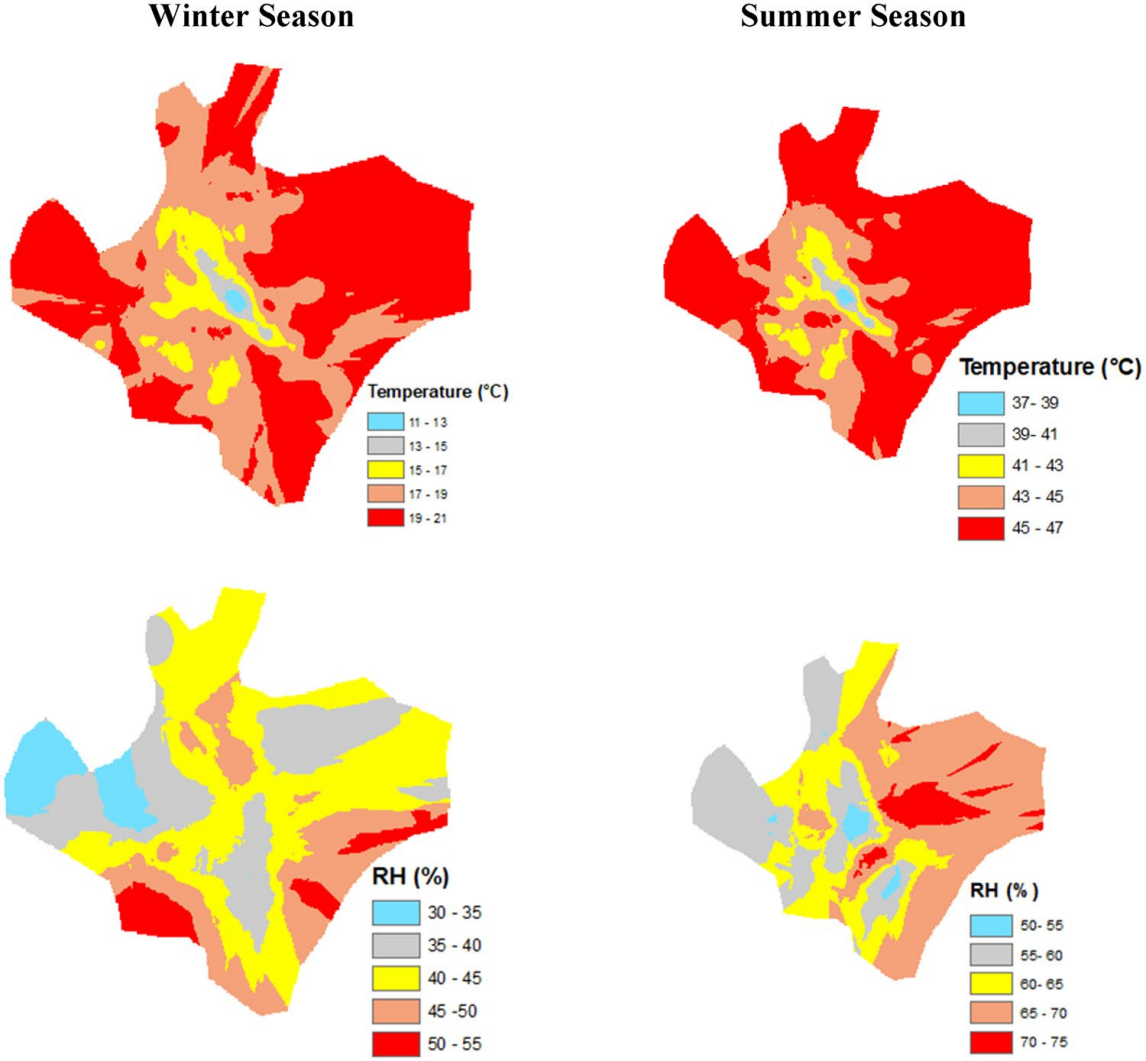
Effect of urban vegetation on temperature and relative humidity in Faisalabad city throughout winter and summer season.

In the urban area of Faisalabad city, the ground level temperature varied from maximum to minimum concentration in the winter and summer seasons. During summer, the temperature peaked at 47 °C in the urban areas. Low values were noticed along the water bodies followed by green vegetation and urban green parks (Fig. 4). Similarly, the winter temperature ranged from 11 to 21 °C at the same locations. In addition, the humidity in the summer season was relatively higher than in winter season. The relative humidity during summer ranged from 37 to 75%, and the moderate humidity in the region of 65% in the southwest of the city has a relatively high vegetation cover. The relative humidity was lower than in summer and ranked 30 < 40 < 55, minimum, moderate, and maximum, respectively in the urban area.

### Noise pollution and green belts analysis

**Figure 5:**
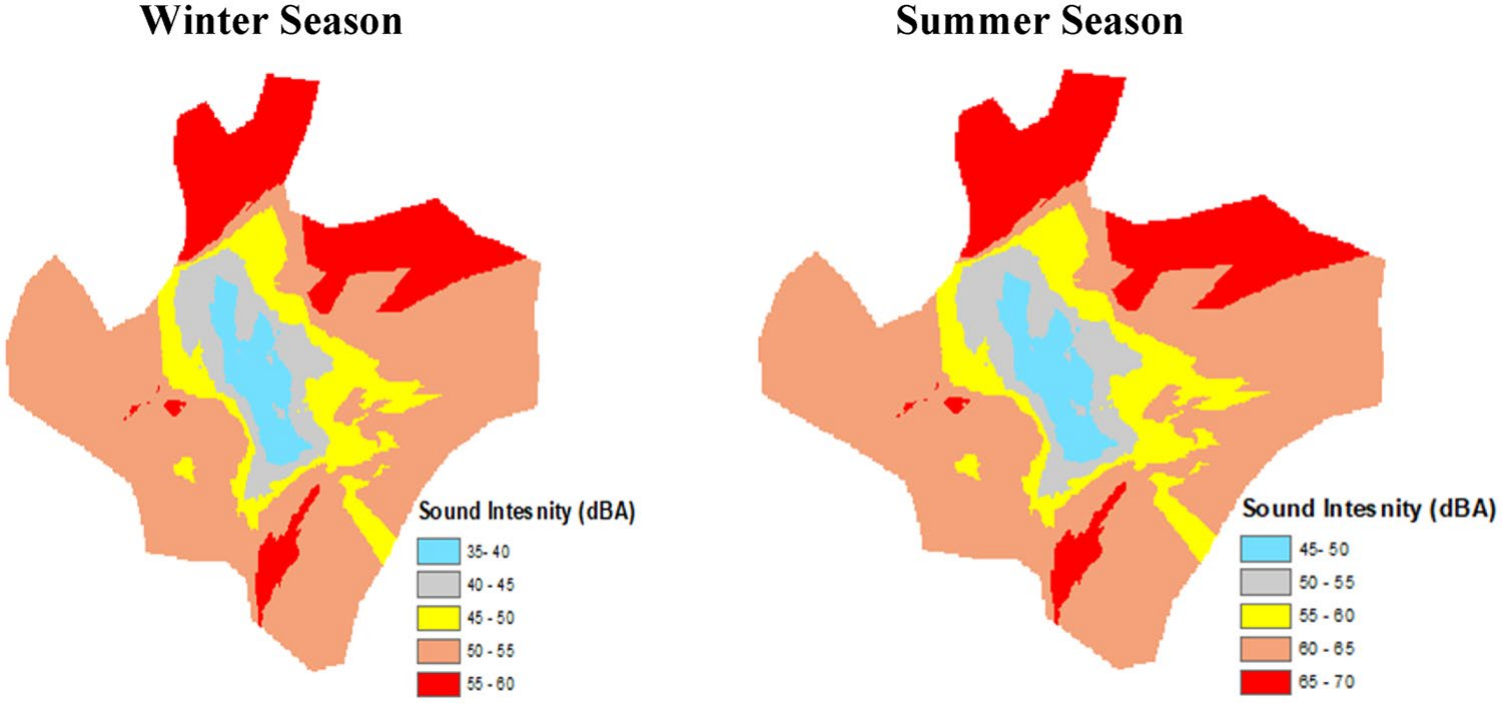
Effect of urban vegetation on sound intensity in Faisalabad city throughout winter and summer season.

The noise pollution recorded in the urban area was found to be different in the winter and summer seasons. In the summer season, the highest value of 60 dBA was in the northeast region of the city, followed by a moderate 55–60 dBA in the central region due to avenue plantation and green space patches in the city. In the city center, the dBA further declined due to the maximum roadside plantation. While in the winter, the dBA was found low due to decreased anthropogenic activities. The maximum and minimum ranges were noise pollution was found between 60 and 35 dBA, respectively. The detail of noise recorded values are illustrated in Fig. 5.

### Correlation and principal component analysis (PCA)

**Figure 6:**
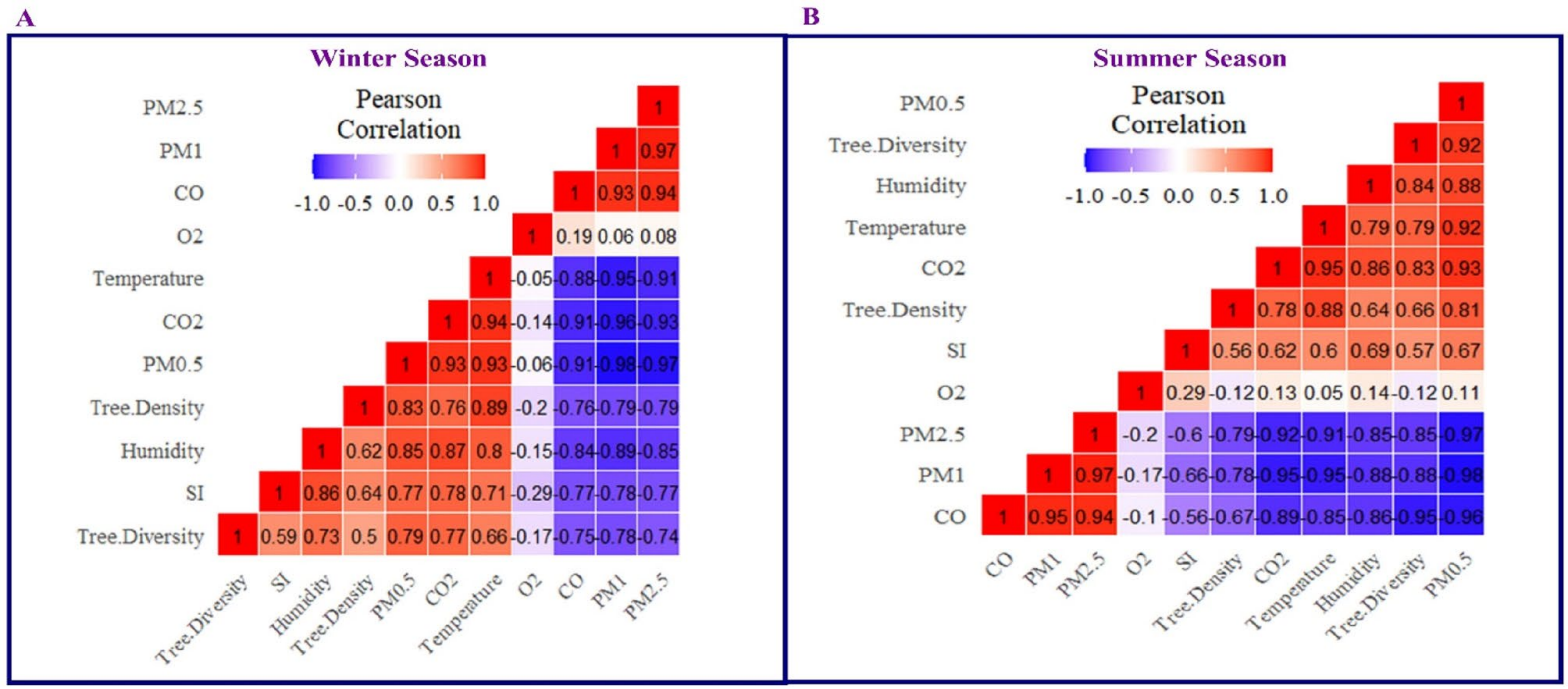
(**A**, **B**) Correlation between atmospheric pollutants in summer and winter season.

Figure 6a represent the correlation between atmospheric pollutants, climatic factors, and urban vegetation during the summer season. The results showed that concentration of carbon monoxide had a strong positive correlation (0.95 & 0.94) with PM_1.0_ and PM_2.5_. While, a similar significant strong correlation (0.95 & 0.93) was observed between CO_2_ concentration with temperature and PM_0.5._ The outcomes exhibited a strong correlation of tree density (0.88 and 0.81) on temperature and PM_0.5_. Whereas a highly negative correlation (− 0.95) was observed between PM_1.0_ on CO_2_ and temperature (Fig. 6A). The results from the winter season present a significant variation in the correlation between atmospheric pollutants, climatic factors, and urban vegetation. Results exhibited a strong positive correlation (0.97) of PM_1.0_ with PM_2.5_, while a similarly strong positive correlation was observed between CO, CO_2,_ and PM_0.5_ with temperature. The results exhibit a positive correlation between tree diversity with humidity, PM_0.5_, CO_2_, tree density, and temperature. However, a strong negative correlation (0.95) between temperature and PM_1.0_ was observed during the winter (Fig. 6B).

**Figure 7:**
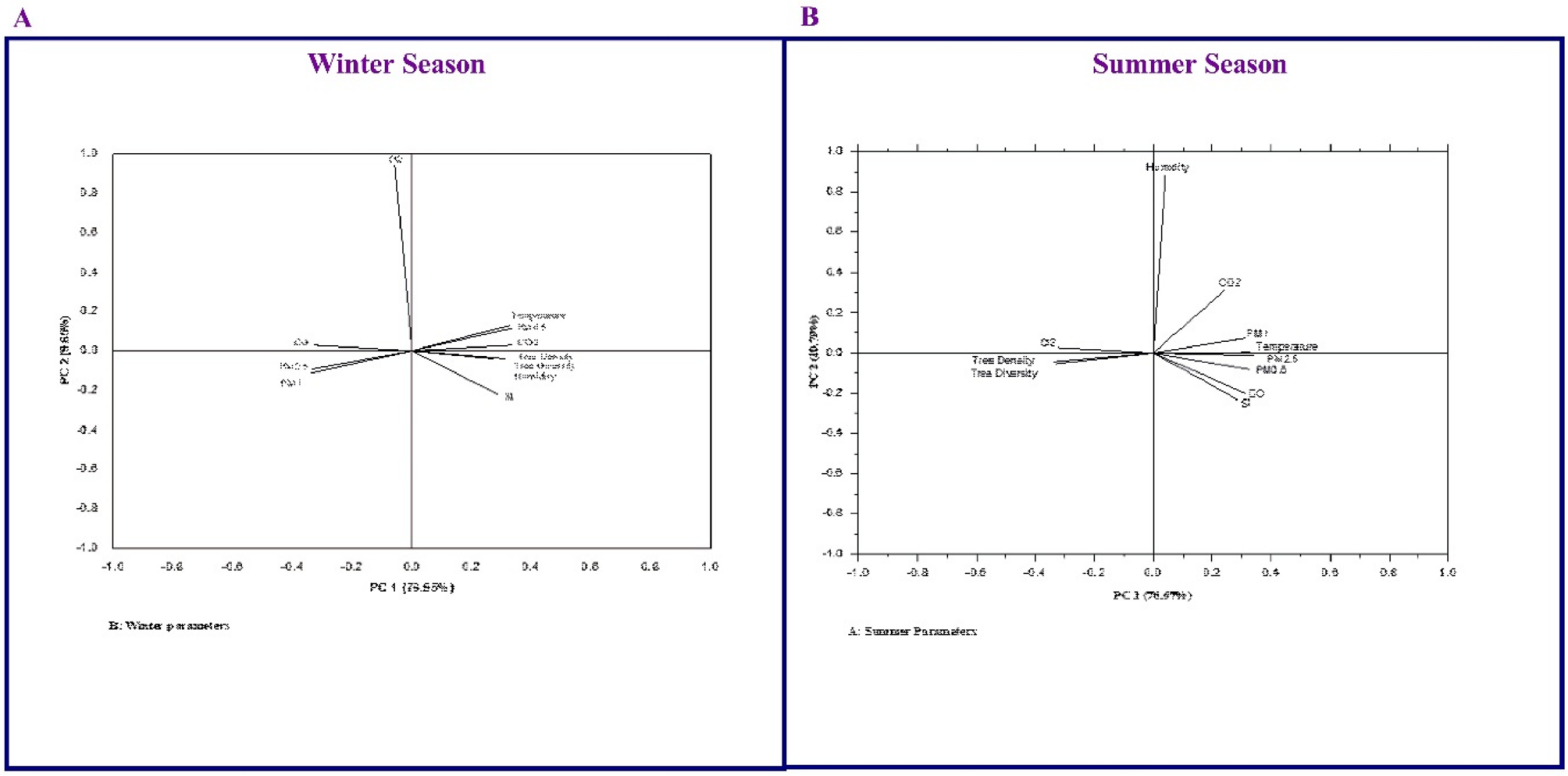
Principal Component Analysis of various atmospheric pollutants measured during the study.

The PCA of different atmospheric pollutants concentration in Faisalabad city in the winter season is presented in Fig. 7A. PCA analysis revealed that eleven principal components (PCs) accounted for 86.63% of the total variation. The temperature, PM_0.5_, CO_2_, tree density, tree diversity, relative humidity, and sound intensity correlated positively, whereas CO, PM_2.5_, and PM_1.0_ correlated negatively with PC1 and accounted for 76.95% of the total variation. CO, PM_2.5_ and PM_1.0_ positively correlated with PC2, whereas temperature, PM_0.5_, CO_2_, tree density, tree diversity, relative humidity, and sound intensity correlated negatively with PC2 and which accounted for 9.68% of the total variation. The different atmospheric pollutants concentrations in the summer season are presented in Fig. 7B. PCA analysis revealed eleven principal components (PCs), which accounted for 87.46% of the total variation. The CO_2_, PM_1.0_, temperature, PM_0.5_, PM_2.5_, CO, and sound intensity correlated positively with PC1, whereas O_2_, tree density and tree diversity correlated negatively with PC1 and accounted for 76.67% of the total variation. O_2_, tree density and diversity correlated positively, whereas CO_2_, PM_1.0_, temperature, PM_0.5_, PM_0.5_, CO, and sound intensity correlated negatively with PC2 and accounted for 10.79% of the total variation.

## Discussion

Pollutant particulate matters, which remain suspended due to buoyancy, are in the sub-micron range, i.e., 10–6 m in diameter^61^. An improved understanding between the associations of particulate morbidity suggests the importance of sub-micron particles (PM_0.5_, PM_1.0_ & PM_2.5_) to which motor vehicles are significant contributors^62^. The average concentration of airborne PM at all the sampling locations is represented in the results. The spatial variations of all measured PM across all the sampling locations were found to be significantly different during the measuring seasons. In this study, the general trend of PM_0.5_, PM_1.0_ & PM_2.5_, CO_2_, CO, and O_2_ were in the order of Winter < Summer (Figs. 4, 5). Overall, the average concentrations of PMs, CO and CO_2_ were found higher in commercial and non-vegetative areas compared to locations with less vegetation. However, the PM concentrations exceeded the permissible limits even in areas with reasonable vegetation as the PM retention capability is different for different leaf surfaces. Furthermore, PM deposition blocks the intensity of sunlight and suppresses the photosynthesis and growth of plants. It also reduces visibility through absorption and scattering by solid and liquid droplets^61^.

The sampling sites with ample vegetative cover also had high traffic densities including heavy-duty diesel vehicles like trucks, buses, vans, etc. Consequently, use of coal and wood for the combustion process and diesel fuel for running electric generators can be the main contributor to PM pollution in the study area^63^. Similar results have been reported previously where high PM concentrations were found in small industrial and commercial centers due to heavy traffic loads, inefficient diesel engines, and poor-quality fuel^64,65^. Apart from this, the suspended road dust and particulate emissions from public and commercial transportation, especially auto-rickshaws and bikes, are also considered as the main contributors to PMs^64,66^. Similarly, higher PM levels at non-vegetative sites were mainly due to the emissions from brick kilns located proximal distance to these sampling points. Some sites like Jinnah Park and Gatwala that were having high vegetation showed low PM due to relatively fewer vehicular and industrial activities.

The overall atmospheric quality of Faisalabad city was found to be very poor, ranging from poor (unhealthy) to very hazardous. The findings of this study indicated that the maximum deteriorated atmospheric quality was recorded around small industries and industrial complexes^67^. Generally, the average atmospheric quality of locations with no or less vegetation was poor than those with high vegetation. The urban areas with higher air quality index indicated that air pollution significantly impacts humans^61^. Similar findings have been reported by Joshi and Swami^68^, where industrial areas are found heavily polluted compared to residential sampling sites in Haridwar, India.

It was noticed that all the studied locations of Faisalabad city were highly polluted. The concentration of PM_0.5_, PM_1.0_ & PM_2.5_, CO_2_, and CO was higher during the summer, while the lowest concentration was measured during winter across all the locations. As all the selected locations share similar environmental conditions, but sources of pollutants like fuel combustion, heavy traffic, and brick kilns had a significant effect on all the airborne particulate concentrations^66^. The concentration of PM_0.5_, PM_1.0_ & PM_2.5_, CO_2_, and CO were found to be higher in commercial and residential areas as compared to green spaces like Jinnah garden site during all three seasons, with maximum values in summer and minimum in winter while the concentration O_2_, relative humidity was higher during winter compared to summer. These findings are consistent with a previous study which showed that the areas with a reasonable number of trees have a particular impact on their ability to reduce air particles^69^. The more significant number of trees and vegetation in the urban areas showed a more remarkable ability to reduce airborne particulate matter^55^. Moreover, proper management, like the pruning of plants in green spaces and residential areas, removes particles from their surface.

This study shows that the monitoring season significantly affected the PM and other airborne particles. The reason behind this could be credited to the interface between the pollution sources of the surroundings and changes in meteorological factors during the particular period^70,71^. Furthermore, the concentration of these particles increased with the wind velocity and relative humidity, thus indicating the effect of weather conditions on the accumulation of these particles in selected sites of Faisalabad city. Similar observations were documented^72,73^, who found a more significant accumulation of particles, including CO_2_ and CO, in urban areas during high relative humidity weather conditions. The result of the present study is also in line with those reported by Zheng et al.^74^ and Zhao et al.^75^, who found higher levels of PM along with CO_2_ and CO in open spaces as compared to green spaces as sometimes the particles absorbed by leaves of different trees prone to bounce back and suspended in the air, thus increasing the concentration of air particles. CO concertation in the current study does not exceed the standard values across all locations during three seasons^76^. These findings are similar to those explained by Rahman et al.^77^, who found less concentration of CO in two cities in China compared to standard values.

The United States EPA has described the acceptable level of noise for road traffic noise as 70 dB. The results of this study indicated that the noise level of all the selected sites of Faisalabad city had exceeded the standard limits. The higher level of noise pollution across all these selected sites is chiefly connected to more significant motor vehicular traffic, mainly the use of vehicle horns, poor maintenance of urban vehicles, etc^78^. These higher levels of noise pollution, as compared to the standard limit, are considerable and can damage the health of exposed individuals in the studied areas^79^. The lowest noise pollution was found in locations with vegetation having less commercial activities and vehicle disturbance. The findings of this study showed that a higher concentration of all measured pollutants and particles was found in industrial areas, followed by residential and vegetative locations. Apart from this, the higher noise levels were also computed in industrial and commercial areas in Faisalabad city compared to vegetative locations. The vegetative areas away from commercial and industrial locations have the lowest concentration of all the measured pollutants. Similar results have been described worldwide by indicating higher concentrations of air pollutants in commercial areas compared to green spaces^80–82^.

## Conclusions

This research analyzed and visualized the linear correlation between atmospheric pollutants, urban vegetation, and meteorological factors in Faisalabad, Pakistan. The outcomes represent that carbon monoxide concentration strongly correlates with PM_1_ and PM_2.5_. At the same time, a highly negative correlation was observed between PM_1_ on CO_2_ and temperature. However, in the case of the winter season, a strong positive correlation of PM_1_ with PM_2.5_ while a similarly strong positive correlation was observed between CO, CO_2,_ and PM_0.5_ with temperature. There is a positive correlation between tree diversity with humidity, PM_0.5_, CO_2_, tree density, and temperature. Arc GIS Analysis distribution atmospheric pollutants maps show that the highest concentrations were near and around poorly vegetated areas. Generally, Arc GIS atmospheric pollutants maps can be used as a basis for the proper distribution of appropriate locations of air pollution measurement stations. Although frequently overlooked, urban vegetation offers various important ecological services to the general population, as listed above. Quantifying these benefits can help establish a foundation for effective urban forest management, reduce cost-to-benefit ratios, and provide residents with a greater understanding of the worth of the natural resources in their communities.

## Data Availability

The authors confirm that the data supporting the findings of this study are available in the article.

## Abbreviations

GIS: Geographic Information System
CO_2_: Carbon dioxide
CO: Carbon monoxide
PPM: Parts per million
PM: Particulate matter
NOx: Nitrous oxides
O_3_: Ozone
RH: Relative humidity
PCA: Principal component analysis
